# A two-step regulatory mechanism dynamically controls histone H3 acetylation by SAGA complex at growth-related promoters

**DOI:** 10.1093/nar/gkaf276

**Published:** 2025-04-10

**Authors:** Sevil Zencir, Daniel Dilg, Maria Jessica Bruzzone, Françoise Stutz, Julien Soudet, David Shore, Benjamin Albert

**Affiliations:** Department of Molecular and Cellular Biology, Université de Genève, 1211, Geneva, Switzerland; Department of Molecular and Cellular Biology, Université de Genève, 1211, Geneva, Switzerland; Department of Molecular and Cellular Biology, Université de Genève, 1211, Geneva, Switzerland; Department of Molecular and Cellular Biology, Université de Genève, 1211, Geneva, Switzerland; Department of Molecular and Cellular Biology, Université de Genève, 1211, Geneva, Switzerland; Department of Molecular and Cellular Biology, Université de Genève, 1211, Geneva, Switzerland; Department of Molecular and Cellular Biology, Université de Genève, 1211, Geneva, Switzerland

## Abstract

Acetylation of histone H3 at residue K9 (H3K9ac) is a dynamically regulated mark associated with transcriptionally active promoters in eukaryotes. However, our understanding of the relationship between H3K9ac and gene expression remains mostly correlative. In this study, we identify a large suite of growth-related (GR) genes in yeast that undergo a particularly strong down-regulation of both transcription and promoter-associated H3K9ac upon stress, and delineate the roles of transcriptional activators (TAs), repressors, SAGA (Spt-Ada-Gcn5 acetyltransferase) histone acetyltransferase, and RNA-polymerase II in this response. We demonstrate that H3K9 acetylation states are orchestrated by a two-step mechanism driven by the dynamic binding of transcriptional repressors (TRs) and activators, that is independent of transcription. In response to stress, promoter release of TAs at GR genes is a prerequisite for rapid reduction of H3K9ac, whereas binding of TRs is required to establish a hypo-acetylated, strongly repressed state.

## Graphical Abstract

**Graphical Abstract:**
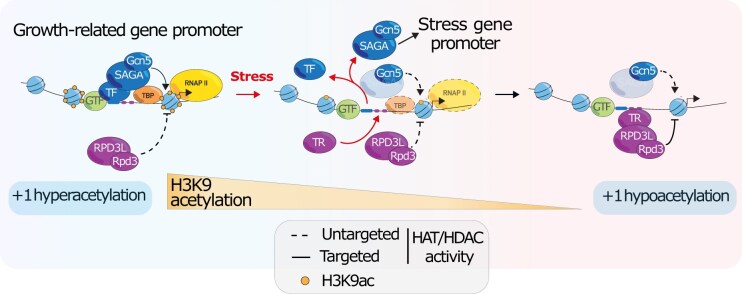

## Introduction

Histone acetylation occurs in distinct combinations, often referred to as the “histone code” [1] and is continuously modulated according to growth conditions by the antagonistic activities of histone acetyltransferase (HAT) and deacetylase (HDACs) enzymes to either promote or block DNA-related processes [2]. General control nonderepressible 5 (Gcn5) is one of the best characterized lysine-specific HATs and is highly conserved from yeast to human [3]. Gcn5 acetylates histone H3 almost exclusively at residues lysine 9, 14, 18, and 23, and its action is generally associated with transcriptional activation. Gcn5 can acetylate nucleosomes as part of two complexes, SAGA (Spt-Ada-Gcn5 acetyltransferase) and a SAGA-like subcomplex called ADA (or the HAT subcomplex), but has also been reported to act independently of these complexes [4–6]. SAGA is also involved in the deubiquitination of histone H2B within coding regions, as well as in pre-initiation complex (PIC) assembly, by facilitating TATA-binding protein (TBP) recruitment to the promoter. Tra1, an essential yeast protein and a common subunit of SAGA and other HAT complexes (most notably NuA4, responsible for H4 acetylation), appears to play a pivotal role by recruiting complexes to promoters through multiple interactions with transcription factors (TFs) [7, 8]. This may allow TFs to act as environmental sensors that target HAT action at specific promoters according to external conditions.

Despite many years of study, the precise role of SAGA in transcription, even in yeast, remains controversial. In one view, SAGA plays a general role in acetylation of H3K9 and transcription initiation for almost all RNA polymerase II (RNAPII) gene promoters [9, 10], whereas an opposing view is that SAGA plays a specific role at a small group of inducible promoters representing ∼8%–13% of RNAPII genes under exponential growth conditions [11–13]. A recent study [14] has highlighted this debate by reporting that ∼80% of RNAPII promoters in yeast are constitutively active and not regulated by the dynamic binding of TFs under any growth conditions, consistent with the finding of minimal SAGA activity at most promoters [11, 13].

Acetylation status of histones at promoters is also regulated by numerous HDACs, including Rpd3, Sir2, Hda1, Hps1, Hos2, and Hos3. Rpd3 is an evolutionary conserved class I HDAC that plays a key role in transcriptional regulation. The absence of Rpd3, but not other HDACs, has been reported to prevent complete gene silencing following inhibition of target of rapamycin complex 1 (TORC1) by rapamycin treatment [15]. Rpd3 plays different roles at the promoter and coding regions due to its ability to exist in two functionally distinct complexes: RPD3L and RPD3S [16, 17]. While RPD3S is involved in diminishing pervasive transcription by deacetylating histones within the coding regions, RPD3L is specifically recruited to promoter regions by various DNA-binding transcriptional repressors (TRs) such as Stb3, Dot6/Tod6, and Xbp1 [15, 18–20]. Importantly, several studies indicate that both HDAC and HAT activities are continuously in equilibrium even in the absence of a specific promoter targeting mechanism [21–25].

The establishment of acetylation states thus entails a complex balance between untargeted and targeted activities of HATs and HDACs that presumably helps cells to adapt their transcription program according to environmental or developmental cues. Given the complexity and often simultaneous occurrence of numerous dynamic events at promoters during fluctuating growth conditions, such as PIC assembly, and binding of TFs and repressors, the determination of cause-and-effect relationships between promoter activities and promoter nucleosome acetylation remains a significant challenge.

Here, we aimed to clarify the mechanisms driving rapid changes in histone H3 acetylation at promoters in cells responding to stress. We began by identifying a large group of growth-related (GR) genes whose promoter H3K9ac state is most strongly regulated by stress and then delineated the roles of transcriptional activators (TAs; Ifh1 and Sfp1), TRs (Stb3/Dot6/Tod6), HATs (primarily Gcn5), and PIC assembly in this response. By carrying out functional studies using deletion mutants or a rapid nuclear depletion system, we revealed the molecular actors as well as the underlying mechanisms driving the rapid transition of promoter-associated nucleosomes at these genes from a H3K9 hyper- to hypoacetylated state. Our results indicate that TAs and repressors play a key role in regulating H3K9ac levels at promoters that is independent of RNAPII activity.

## Materials and methods

### Yeast strains and growth conditions

All experiments presented in this study were performed using the budding yeast *Saccharomyces cerevisiae* (*S. cerevisiae*). A complete list of strains used in this study is provided in Supplementary Table S5. Strains were generated by genomic integration of tagging or disruption cassettes as described [26, 27]. The strain overexpressing Crf1 was obtained by deleting *IFH1* in a strain carrying TAP-tagged *CRF1* driven by the strong *GPD1* promoter on a multi-copy plasmid (pRS-425-GPD; Addgene plasmid #14274), which rescues the lethality of *ifh1*Δ. The untagged YDS2 strain was used as a wild-type (WT) control. For Chromatin immunoprecipitation (ChIP) of Rpb1 in the TBP anchor-away strain Fig. 4G–J, cross-linked chromatin obtained from fission yeast *Schizosaccharomyces pombe* (*S. pombe*) was used as a spike-in control. Yeast cells were grown in YEP medium (1% yeast extract and 2% peptone) supplemented with 2% glucose as carbon source (YEPD) at 30°C overnight and then diluted to OD_600_ = 0.1. Experiments were performed with exponential phase cells harvested between an OD_600_ of 0.4 and 0.6, unless otherwise indicated.

### Protein depletion experiments

The anchor-away system was used for conditional depletion of target proteins as described [28]. Log-phase growing cells (OD_600_ = 0.3–0.4) were treated with 1 μg/ml rapamycin (resuspended in 90% ethanol and 10% Tween-20) before collection of cells. For TBP anchor-way, cells were treated with rapamycin for 30 min. For Sfp1 and Ifh1 single and double anchor-way, cells were treated with rapamycin for 60 min.

### Rapamycin treatment for stress experiments

For stress experiments, cells were incubated in YEPD until log phase at 30°C, then the samples were treated with rapamycin at a final concentration of 200 nM for 20 min to inhibit the growth regulator TORC1. The cells were collected following rapamycin treatment at the indicated time points before crosslinking.

### ChIP-Seq

For ChIP-seq of Rpb1, 50 ml of yeast cultures; for H3K9ac ChIP-seq experiments, 300 ml of yeast cultures; for Stb3 and Rpd3 ChIP-seq experiments, 150 ml of yeast cultures in YEPD were collected at OD_600_ = 0.4–0.6 for each condition. The cells were crosslinked with 1% formaldehyde for 10 min and quenched by adding 125 mM glycine for 5 min at room temperature. The ChIP protocol was performed as previously described [29]. Briefly, crosslinked cells were lysed by bead beating, chromatin was sonicated, and the soluble fraction was incubated with the appropriate antibodies for 1 h at 4°C. Magnetic beads coupled to IgG against rabbit or mouse (Invitrogen, Dynabeads^™^ M-280 Sheep Anti-Rabbit or Anti-Mouse IgG) were added to the samples and incubated for an additional 3 h at 4°C. A defined amount (5% of total chromatin) of crosslinked and sonicated chromatin from *S. pombe* was added to *S. cerevisiae* chromatin prior to the immunoprecipitation step. ChIP was performed using the following antibodies: anti-histone H3 (Abcam ab1791), anti-H3K9ac (Millipore 07-352), anti-RNAPII (Abcam ab5131), anti-Flag (Sigma F3165), and anti-Myc (a homemade antibody clone 9E10). The DNAs were purified using the MinElute PCR Purification Kit (Qiagen, 28006), and ChIP-seq libraries were prepared using TruSeq ChIP Sample Preparation Kit (Illumina, IP-202-1012), according to manufacturer’s specifications. The libraries were sequenced in single-end mode on an Illumina HiSeq 2500 at the IGE3 Genomics Platform of the University of Geneva (https://ige3.genomics.unige.ch/).

### ChIP-seq quantification

RNAPII (Rpb1)-binding signal was quantified in the transcribed region of all genes with well-determined transcription start site (TSS) and transcription termination site (TTS) as described [29]. ChIP-seq peaks of Ifh1, Stb3, and Rpd3 binding were defined by shifting the plus and minus strand ChIP-seq profiles towards each other by 150 bp and extending each read by 40 bp. To quantify ChIP-seq signals for each promoter, a ratio between the total number of reads from each sample in a 400 bp region upstream the TSS [30] of each open reading frame (ORF) and the total number of reads from the same region obtained with mock IP of the control strain. To compare depleted versus nondepleted cells, we divided the signal from the +rapamycin (+Rap) samples by the signal from the −rapamycin (vehicle) samples and log2 transformed this value. All data were mapped using the Galaxy server [31].

### Motif search

Motif search in the promoters (defined as the 400-bp-long sequence upstream of the TSS) was performed using the DREME algorithm [32] (https://meme-suite.org/meme/tools/meme, classic mode, Zero or One Occurrence per Sequence, Minimum width 6, and Maximum width 20). Motif identification for known DNA-binding proteins was performed using the TOM algorithm [33].

### Heat maps, plots, and statistics

The heat maps were generated by using the Galaxy server (https://usegalaxy.org/; [31]. The scatter plots and box blots were generated by using Prism10.0 (GraphPad). In all box plots, the box shows the 25th–75th percentile, whiskers show the 10th–90th percentile, and dots show the 5th and 95th percentiles. *R*-values for all correlation measurements are the Pearson’s correlation coefficients. Statistical significance of difference between groups was evaluated using the ordinary one-way analysis of variance (ANOVA) test.

## Results

### Identification of promoters shifting from H3 hyper- to hypo-acetylated state upon stress

**Figure 1.**
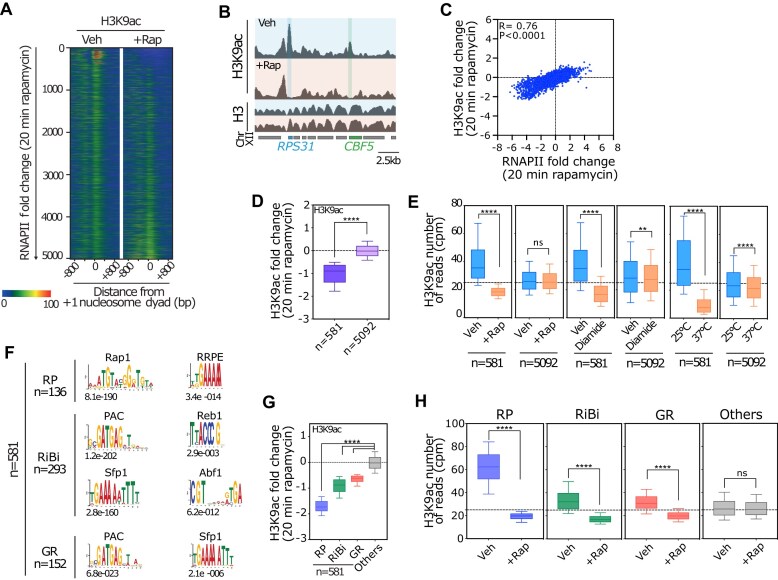
Promoters of GR genes are the major targets of rapid stress-induced H3K9 deacetylation. (**A**) Heat maps showing H3K9ac ChIP-seq signals of 5673 yeast genes aligned to +1-nucleosomes in WT cells treated (+Rap) versus nontreated (Vehicle) with rapamycin for 20 min. Heat maps are sorted by increasing log2 fold change RNAPII signal over the complete open reading frame of all protein-coding genes upon rapamycin treatment. The average signal for H3K9ac was quantified in a window of 75 base pairs (bp) centered on the +1-nucleosome. Signals for a window of −800 to +800 bp relative to +1 nucleosome are displayed (*x*-axis). H3K9ac ChIP-seq data are representative of *n* = 2 ChIP-seq experiments. (**B**) Genome browser tracks showing H3K9ac and H3 ChIP-seq read counts on chromosome XII in WT cells treated (+Rap) versus nontreated (Vehicle) with rapamycin for 20 min. The position of individual genes are shown below the tracks. H3K9ac and H3 data are representative of *n* = 2 ChIP-seq experiments. RNAPII ChIP-seq data are representative of *n* = 3 ChIP-seq experiments. (**C**) Scatter plot showing the correlation between the change in acetylation (*y*-axis, as measured by H3K9ac ChIP-seq) and transcription (*x*-axis, as measured by Rpb1 ChIP-seq) in WT cells, calculated as a log2 fold chance between the signal in rapamycin treated versus nontreated cells (+Rapamycin/−Rapamycin). Each dot represents a gene (4966 in total). The average signal for RNAPII was quantified from the TSS to the TTS. The average signal for RNAPII and H3K9ac was quantified as in panel (A). *R*-values for correlation measurements are the Pearson’s correlation coefficients (*R* = 0.76, ****P< 0.0001). H3K9ac and RNAPII ChIP-seq data are representative of *n* = 2 and *n* = 3 ChIP-seq experiments, respectively. (**D**) Box plot showing H3K9ac change in WT cells treated with rapamycin (200 nm final concentration) for 20 min for indicated 581 genes. The genes were isolated using a stringent cut-off where they exhibit both downregulation (log2 fold change for Rpb1 ChIP-seq signal < −1) and deacetylation (log2 fold change for H3K9ac ChIP-seq signal < −0.41). The change in H3K9ac calculated as log2 fold change between the signal in +Rapamycin/−Rapamycin-treated cells. Statistical significance of difference between two groups was evaluated using the Mann–Whitney test (****P< 0.0001). (**E**) Box plots showing total number of mapped reads for H3K9ac ChIP-seq in WT cells treated or nontreated either with rapamycin (Vehicle versus +Rap), diamide (Vehicle versus Diamide) and heat shock (25°C versus 37°C) stresses for indicated gene categories. Data for “Diamide” stress and “Heat shock” stress were obtained from Weiner *et al.* [82] and Mittal *et al.* [13], respectively. The genes are annotated according to treatment: nontreated samples (Vehicle); treated samples (+Rap/Diamide/Heat shock). Statistical significance of difference between two groups was evaluated using the Mann–Whitney test (****P< 0.0001; **P< 0.05; ns: nonsignificant). (**F**) Enrichment of motifs in the promoters of each gene cluster (RP, RiBi, and GR) that undergoes rapid transcriptional downregulation and promoter H3K9 deacetylation following 20 min of rapamycin treatment. *E-*values for each motif, name of the motif (PAC and RRPE) or associated TFs (Rap1, Reb1, Abf1, and Sfp1) are indicated. (**G**) Box plot showing H3K9ac change (as measured by H3K9ac ChIP-seq) in WT cells treated or nontreated with rapamycin for 20 min for indicated gene categories (5673 in total). The genes are annotated according to functional groups as RP, RiB , GR, and all other genes. The change in H3K9ac calculated as log2 fold change between the signal in +Rapamycin/−Rapamycin treated cells. Statistical significance was defined by ordinary one-way ANOVA test for multiple groups (****P< 0.0001). (**H**) Box plots showing total number of mapped reads for H3K9ac ChIP-seq in WT cells treated or nontreated with rapamycin (Vehicle versus +Rap) for indicated gene categories. The genes are annotated according to functional groups as RP, RiBi, GR, and all other genes. Statistical significance of difference between two groups was evaluated using the Mann–Whitney test (****P< 0.0001; ns: nonsignificant).

To understand regulatory mechanisms governing histone H3 acetylation dynamics at promoters, we treated cells with rapamycin, a drug that specifically inhibits the master growth-stimulating kinase TORC1 on a time scale of minutes. We chose 20 min of rapamycin treatment because the downregulation of ribosomal protein (RP) and ribosome biogenesis (RiBi) gene expression, as measured by RNAPII association with their gene bodies, is close to its maximum at this timepoint, but only partial after 5 min (Supplementary Fig. S1A). Consistent with this finding, messenger RNA (mRNA) levels are already strongly affected by 20 min (Supplementary Fig. S1B), a time sufficient to induce significant changes in the phosphorylation state and nuclear localization of key transcriptional regulators (both activators and repressors; [34, 35]). We observed changes in the H3K9ac pattern at numerous promoters (Fig. 1A and B, and Supplementary Fig. S1C), which were closely correlated with changes in RNAPII recruitment (Fig. 1C). Using a stringent cut-off (see “Materials and methods” section), we identified a subset of 581 promoters (Supplementary Table S1), controlling >10% of all RNAPII-transcribed genes, in which both recruitment of RNAPII and H3K9ac levels were strongly reduced following rapamycin treatment (Fig. 1D, and Supplementary Fig. S1C and D). The same effect was observed under other stress conditions (heat shock, oxidative stress, and carbon withdrawal; Supplementary Fig. S1E–G), or as synchronized cells transition from the oxidative to reductive building/charging phases of the metabolic cycle (Supplementary Fig. S1H). The strong decrease in H3K9ac under these conditions cannot be explained by a local decrease of total H3 histone at these promoters (Supplementary Fig. S1I–L), whose −1 and +1 nucleosome positions are essentially unchanged following stress (Supplementary Fig. S1M and N and in [13, 14]). Furthermore, the rapid deacetylation at these promoters is not limited to H3K9ac, as we also observed a rapid reduction of acetylation at lysine 4, 14, 18, 23, 27, and 56 residues on histone H3 following diamide treatment (Supplementary Fig. S1O). Importantly, these promoters exhibit the highest level of acetylation during exponential growth compared to all other promoters, yet they undergo a rapid transition to the lowest level of acetylation upon stress, suggesting the existence of specific mechanisms regulating these two opposing acetylation states (Fig. 1E).

As a first step towards identifying the molecular actors regulating the acetylation state of these 581 promoters, we noted that their linked genes fall into three distinct but related categories: RP genes, RiBi genes and a third group of genes enriched in GR functions (Fig. 1F; Supplementary Fig. S1P, and Supplementary Tables S2 and S3). Promoters of RP and RiBi genes bind insulator TFs such as Rap1 and Abf1, which facilitate the establishment of nucleosome-depleted or nucleosome-free regions and remain stably associated at promoters under stress [14, 37–40]. In contrast, the two stress-responsive TFs Ifh1 [35, 41–44] and Sfp1 [45–47] stimulate expression of RP and RiBi genes, respectively, and are both rapidly released from promoters following stress [29, 39, 41, 48]. Accordingly, Ifh1 depletion strongly affected RNAPII recruitment at RP genes while depletion of Sfp1 revealed a similarly strong effect on RiBi genes, and a slight effect on GR genes, suggesting that this latter group of genes could be regulated, at least partially, by Sfp1 (Supplementary Fig. S1Q). Notably, these three groups of genes have different levels of H3K9ac at their promoters before stress yet arrive at a very similar level of hypoacetylation following TORC1 inactivation, suggesting that a common mechanism may mediate their rapid and complete deacetylation (Fig. 1G and H). Indeed, *in silico* analysis reveals that these three groups of promoters are enriched in specific binding motifs termed RRPE (TGAAAA) and PAC (GATGAG) [49–51], known to promote the recruitment of the TRs Stb3 and Dot6/Tod6 [52–54], respectively, upon TORC1 inactivation (Fig. 1F). The key TAs (Ifh1 and Sfp1; [55–58]) described above have been reported to interact with HAT complexes whereas the TRs (Stb3, Dot6, and Tod6; [59, 60]) have been shown to interact with the RPD3 HDAC. Consistent with this latter point, we and others have shown that the absence of Rpd3 almost completely abolishes H3K9 deacetylation following rapamycin treatment or carbon starvation (Supplementary Fig. S1R and [61]). These data suggest that multiple interactions between TFs and either HAT or HDAC complexes may explain the specific ability of RiBi, RP and GR gene promoter nucleosomes to switch between hyper- and hypo-acetylation states in a growth-dependent manner.

### H3K9ac at promoters of growth-related genes is dependent on SAGA activity

**Figure 2.**
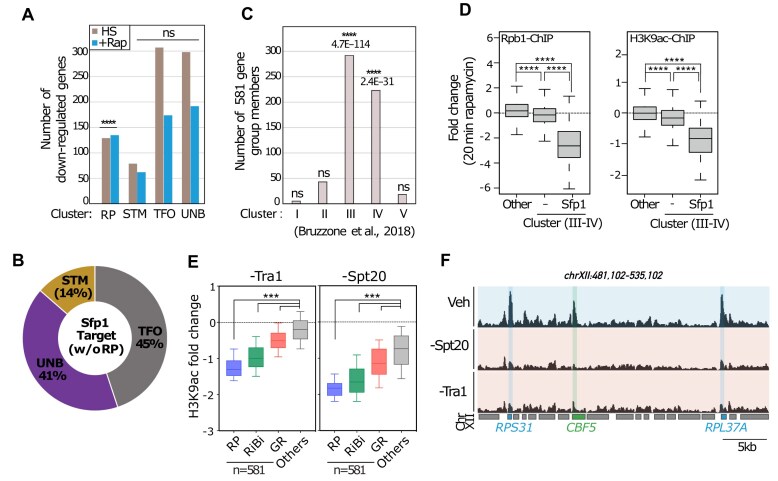
H3K9ac at promoters of GR genes is dependent of SAGA activity. (**A**) Bar plot showing the number of the gene groups downregulated upon heat shock (HS) and TORC1 inactivation (+Rap) treatment in indicated gene clusters categorized according to [14]. The change in RNAPII calculated as log2 fold change between the signal in heat shock treated (37°C/25°C) and rapamycin treated (+Rapamycin/−Rapamycin) cells. (**B**) Pie chart showing the percentage of the genes for STM (14%), TFO (45%), and UNB (41%) gene categories in Sfp1-target genes (*n* = 441, without RP genes) defined in [45]. (**C**) Bar plot showing the number of the GR genes (*n* = 581) in different clusters (clusters I–V) defined by [66], based on their expression defects following depletion of either Gcn5, Esa1, Med17, or Tra1 (****P< 0.0001; ns: nonsignificant). (**D**) Box plots showing the log2 fold change in Rbp1 occupancy (left panel) and H3K9ac level (right panel) in WT upon 20 min rapamycin stress for genes in clusters III and IV (*n* = 1674) and all others (*n* = 3332). Cluster III and IV are categorized based on whether they contain Sfp1-target genes (Sfp1, *n* = 554) or not (−, *n* = 1121). The average signal for RNAPII was quantified from the TSS to the TTS. The average signal for H3K9ac was quantified in a window of 75 bp centered on the +1 nucleosome. H3K9ac and RNAPII ChIP-seq data are representative of *n* = 2 and *n* = 3 ChIP-seq experiments, respectively. (**E**) Box plots showing the log2 fold change in H3K9ac ChIP-seq signal in Tra1-depleted (−Tra1, left panel) and Spt20-depleted (−Spt20, right panel) cells [data from Mittal *et al.*, 2022; an auxin-induced degradation (AID) system was used for depletion, in which a D-tag was fused to the C-terminus of the target protein. The tagged factors were degraded by treating with 1 mM indole-3-acetic acid for 30 min]. The genes are annotated according to functional groups as RP, RiBi , GR , and all other genes. Statistical significance for multiple groups was defined by ordinary one-way ANOVA using Dunnett’s multiple comparison test with “others” group as a control (***P< 0.0005). (**F**) Snapshots of genome browser tracks showing a region of chromosome XII for H3K9ac ChIP-seq signal in WT, Spt20- or Tra1-depleted cells (data from [13]). The position of *RPS31*, *RPS37A*, and *CBF5* are annotated below the tracks. H3K9ac ChIP-seq data are representative of *n* = 2 ChIP-seq experiments.

To uncover cofactors (e.g. HATs, HDACs, and TFIID) involved in regulation of these genes, we first turned to a comprehensive dataset reported in two recent studies describing all RNAPII promoter architectures and their different modes of regulation [13, 14]. Based on the analysis of genome-wide ChIP-exo experiments carried out on nearly 400 proteins, these authors proposed that at least 80% of RNAPII-transcribed genes have a homogeneous organization and are constitutively expressed at low/moderate levels in all conditions. These constitutively expressed genes were divided into two groups: UNB (unbound by TFs or coactivators) and TFO (TF only, lacking coactivators). The remaining genes, considered “inducible,” were also divided into two groups: RP genes and STM (SAGA, TUP, and/or Mediator/SWI-SNF (SWItch/Sucrose Non-Fermentable)) genes, both classified as SAGA-dependent. Considering their similar ability to be rapidly downregulated and deacetylated following rapamycin treatment, we imagined that RiBi and other GR genes might share a similar set of cofactors with RP genes. However, we note that most of these genes were assigned to the constitutive UNB or TFO categories (Fig. 2A), which is surprising given that genes related to RiBi, transfer RNA (tRNA) processing and translation have been described as prominent members of the “environmental stress response,” initially characterized in yeast [62–64].

One possible reason for the classification of many RiBi and GR genes in the unregulated UNB and TFO categories is that ChIP-exo fails to detect Sfp1, a key TF acting at many of these genes, whose binding is robustly detected by Chromatin Endogenous Cleavage-sequencing (ChEC-seq; [45, 65], and noted in Supplementary Fig. S2A and Supplementary Table S4). This may in part explain why the majority of non-RP Sfp1 target genes are classified as constitutive UNB or TFO genes (Fig. 2B).

Given the apparent limitations of ChIP-exo with respect to TF and co-activator analysis of RiBi and GR promoters, we turned to a meta-analysis of transcription following either Gcn5 (SAGA-associated), Esa1 (NuA4-associated), Med17 (Mediator-associated), or Tra1 (SAGA- and NuA4-associated) depletion, in which genes were clustered according to their expression defect to identify coactivator involvement [66]. Interestingly, ∼90% of the 581 genes we identified as rapidly and strongly deacetylated following TORC1 inhibition belong to two specific groups of genes in the Bruzzone *et al.* [66] analysis: clusters III (*n* = 712) and IV (*n* = 962), that were both shown to be regulated by Gcn5, presumably through SAGA and/or ADA complexes (Fig. 2C). Notably, cluster III contains nearly all the RiBi and GR genes, whereas cluster IV contains nearly all the RP genes. As pointed out above, both RiBi and RP genes are regulated by Sfp1, whereas nearly all RPs are strongly Ifh1 dependent. Interestingly, most of the other genes in these two clusters are not as strongly affected by rapamycin-induced stress (either at the level of H3K9 or RNAPII decrease) as those in the two clusters where Sfp1 binding is detected (Fig. 2D). This observation suggests that the rapid release of Sfp1 and/or Ifh1 [41, 45, 47, 67, 68] might be a key determinant of the rapid reduction of H3K9ac at the promoters of these genes (see below).

To determine the roles of the SAGA and ADA complexes in H3K9 promoter acetylation at these genes exploited published data from depletion or deletion experiments of Spt20, Tra1, and Ahc1 carried out in unstressed cells [13]. We found that nuclear depletion of either Spt20 or Tra1 led to rapid reduction of H3K9ac at RP, RiBi, and GR genes, suggesting that continuous activity of SAGA is required to maintain acetylation at these promoters (Fig. 2E and F). This effect was not limited to H3K9ac, since we also observed that H3K18ac is decreased at RP, RiBi, and GR genes upon deletion/depletion of other SAGA subunits ([69]; Supplementary Fig. S2B). On the other hand, deletion of Ahc1 (affecting only the ADA complex) did not trigger a decrease in H3K9ac (Supplementary Fig. S2C and D) indicating that Gcn5 is targeted to these promoters via the SAGA complex, and not through ADA. Notably, even though the promoters of these three gene groups display the highest SAGA-dependent H3K9ac levels in rapidly growing cells and the lowest following stress, Gnc5 depletion has only a modest effect on their expression in unstressed cells, due at least in part to redundancy with Esa1, the NuA4-associated HAT ([66]; Supplementary Fig. S2E).

### Rapid reduction of H3K9ac following stress depends on activator release, whereas establishment of a hypoacetylated state requires repressor binding

**Figure 3.**
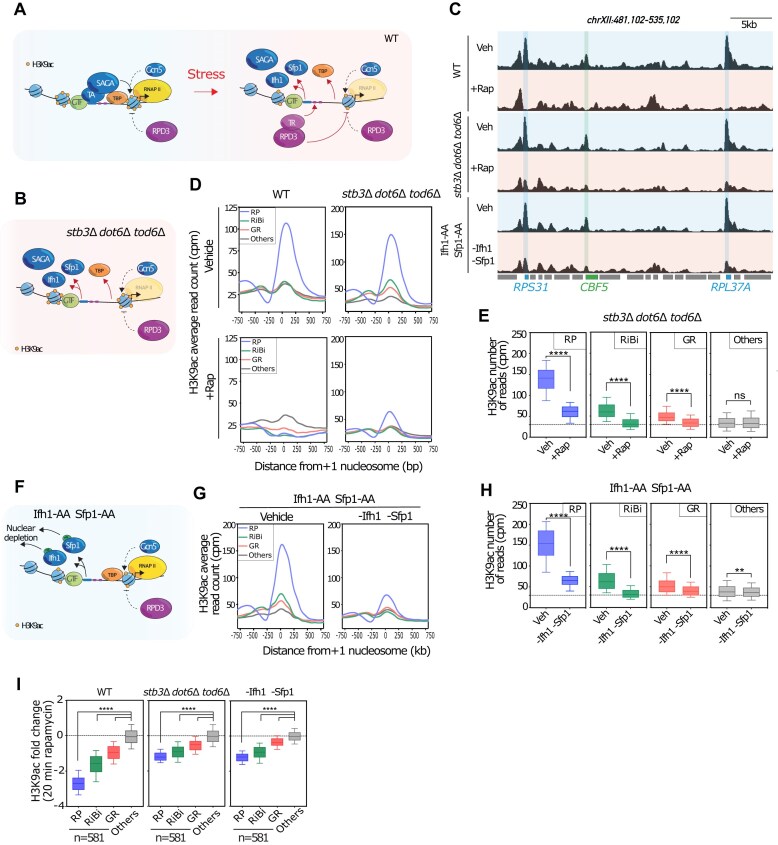
Rapid reduction of H3K9ac is dependent on the release of TAs. (**A**) Schematic representation of factors that might play a role in altering H3K9ac state and RNAPII recruitment at the promoters of GR genes upon stress; rapid release of TAs and their associated cofactors (SAGA), binding of TRs which promote Rpd3 (HDAC) recruitment and a drastic reduction of RNAPII activity. (**B**) Schematic representation of the experimental outline to test the effect of TRs on H3K9 acetylation; Stb3, Dot6, and Tod6 deleted strain (*stb3*Δ*dot6*Δ*tod6*Δ) was treated with 200 nM rapamycin for 20 min. Cells were harvested for cross-linking and H3K9ac ChIP-seq following rapamycin (or vehicle control) treatment. (**C**) Snapshots of sample genomic region of chromosome XII showing H3K9ac ChIP-seq signal in WT cells and in *stb3*Δ*dot6*Δ*tod6*Δ mutant treated without/with rapamycin (Veh/+Rap), and in Sfp1 and Ifh1 nondepleted (veh)/double-depleted cells (-Sfp1 -Ifh1) shown from top to bottom panels, respectively. The positions of *RPS31*, *RPS37A*, and *CBF5* are annotated below the tracks. H3K9ac ChIP-seq data in WT cells and in *stb3*Δ *dot6*Δ *tod6*Δ mutant triple mutant cells treated without/with rapamycin (Veh/+Rap), and in Sfp1 and Ifh1 depleted cells are representative of *n* = 2 ChIP-seq experiments. (**D**) Average H3K9ac signal centered on the +1-nucleosome dyad across all genes (5673 in total) following 20 min rapamycin treatment or nontreated in both WT (left panel) and in *stb3*Δ*dot6*Δ*tod6*Δ mutant (right panel) cells. Genes are categorized based on functional groups: RP, RiBi, GR , and all other genes. H3K9ac ChIP-seq data in WT cells and in an *stb3*Δ *dot6*Δ *tod6*Δ mutant treated without/with rapamycin (Veh/+Rap) are representative of *n* = 2 ChIP-seq experiments. (**E**) Box plots showing total number of mapped reads for H3K9ac ChIP-seq in *stb3*Δ *dot6*Δ *tod6*Δ cells treated or nontreated with rapamycin (Vehicle versus + Rap) for gene categories of RP, RiBi, GR, and all other genes. Statistical significance of difference between two groups was evaluated using the Mann–Whitney test (****P< 0.0001; ns: nonsignificant). H3K9ac ChIP-seq data in an *stb3*Δ *dot6*Δ *tod6*Δ mutant treated without/with rapamycin (Veh/+Rap), and in Sfp1 and Ifh1 depleted cells are representative of *n* = 2 ChIP-seq experiments. (**F**) Schematic representation of the experimental outline to test the effect of TFs on H3K9 acetylation; Ifh1 and Sfp1 were fused to the rapamycin-binding domain FRB. An Ifh1-FRB Sfp1-FRB strain was treated with 1 μg/ml rapamycin for 60 min to induce Ifh1 and Sfp1 double nuclear depletion. Cells were harvested for cross-linking and H3K9ac ChIP-seq following to rapamycin (or vehicle control) treatment. (**G**) Average plot showing H3K9ac signal centered on the +1-nucleosome dyad across all genes (5673 in total) following double depletion of Sfp1 and Ifh1. Genes are categorized as in panel (D). H3K9ac ChIP-seq data in Sfp1 and Ifh1 depleted cells are representative of *n* = 2 ChIP-seq experiments. (**H**) Same as panel (E) for H3K9ac ChIP-seq in Ifh1- and Sfp1-depleted cells compared to vehicle cells (Vehicle versus -Ifh1 -Sfp1) for gene categories of RP, RiBi, GR, and all other genes. Statistical significance of difference between two groups was evaluated using the Mann–Whitney test (****P< 0.0001, **P< 0.05). H3K9ac ChIP-seq data in Sfp1- and Ifh1-depleted cells are representative of *n* = 2 ChIP-seq experiments. (**I**) Fold change of H3K9ac ChIP-seq signal in WT, in *stb3*Δ *dot6*Δ *tod6*Δ and in Ifh1- and Sfp1-depleted cells, treated or nontreated with rapamycin and plotted for the gene categories RP, RiBi, GR, and all other genes. The change in H3K9ac was calculated as log2 fold change between the signal for rapamycin-treated and untreated cells (+Rapamycin/–Rapamycin; 20 min) for WT and *stb3*Δ *dot6*Δ *tod6*Δ mutant cells, and upon 60 min depletion of Ifh1 and Sfp1. Statistical significance for multiple groups was defined by ordinary one-way ANOVA using Dunnett’s multiple comparison test with “others” group as a control (***P< 0.0005). ChIP-seq data following double depletion Sfp1 and Ifh1 or signal in *stb3*Δ*dot6*Δ*tod6*Δ cells are representative of *n* = 2 ChIP-seq experiments.

Although we identified TAs, repressors, and their associated cofactors linked to GR promoters, the concurrence of multiple changes observed at these promoters under stress—such as rapid release of TFs (Ifh1 and Sfp1) and their associated cofactors (SAGA), binding of repressors (Stb3, Dot6, and Tod6), and a drastic reduction of RNAPII activity—obscures the cause-and-effect relationship between these distinct processes and H3K9ac state at the +1 nucleosome (Fig. 3A). To overcome this difficulty, we designed an experimental set-up to study separately the influence of TRs and activators.

We first examined the change in H3K9ac upon stress in the absence of Stb3, Dot6, and Tod6, three well characterized repressors of these GR genes (Fig. 3B). In *stb3*Δ *dot6*Δ *tod6*Δ triple-mutant cells, it has been shown that the mRNA levels of GR genes are downregulated less efficiently following inhibition of TORC1 downstream effector Sch9 ([34]; see also Supplementary Fig. S3). Following rapamycin treatment, we still observed a rapid decrease in H3K9ac at the +1 nucleosome, indicating that this phenomenon is independent of these targeted repressors (Fig. 3C–E). Nevertheless, we found that the triple mutant fails to completely establish a hypo-acetylated state at the promoters (Fig. 3E). These data demonstrate that repressors are required to strongly ablate H3K9ac at the +1-nucleosome following stress, whereas rapid reduction of H3K9ac can be achieved by another process, likely linked to the release of TFs and their associated co-factors observed during stress.

We therefore directed our attention towards the two TAs of these genes, Ifh1 and Sfp1 (Fig. 3F), whose release might be sufficient to induce a rapid reduction of H3K9ac if they promote HAT recruitment. Consistent with this idea, we found that nuclear depletion of Sfp1 and Ifh1 triggers a strong decrease in H3K9ac (Fig. 3G and H) though, interestingly, neither depletion fully mimics the hypo-acetylation of H3K9ac observed upon stress (Fig. 3G and H). Notably, this intermediate acetylation state is like that observed in *stb3*Δ *dot6*Δ *tod6*Δ cells treated with rapamycin (Fig. 3I). Thus, in both conditions promoter release of Sfp1 and Ifh1 (either through TORC1 inhibition or cytoplasmic anchoring) triggers a strong decrease in H3K9ac but is not sufficient to achieve a hypo-acetylated state in the absence of TRs. Importantly, even though H3K9ac can also be deacetylated by HDACs other than Rpd3, we show that absence of Rpd3 nearly abolishes H3K9ac following stress, confirming that histone deacetylation at these promoters is mainly dependent on Rpd3.

These data are consistent with the existence of two independent processes involving both the release of TAs, which leads to a rapid decrease in H3K9ac, and the binding of repressors, which are essential to establish the hypoacetylation state. The rapid and strong reduction of H3K9ac observed in WT cells upon stress requires the combination of these two processes.

### Release of TFs, rather than reduction of RNAPII activity, is the first necessary step for rapid deacetylation of H3K9

**Figure 4.**
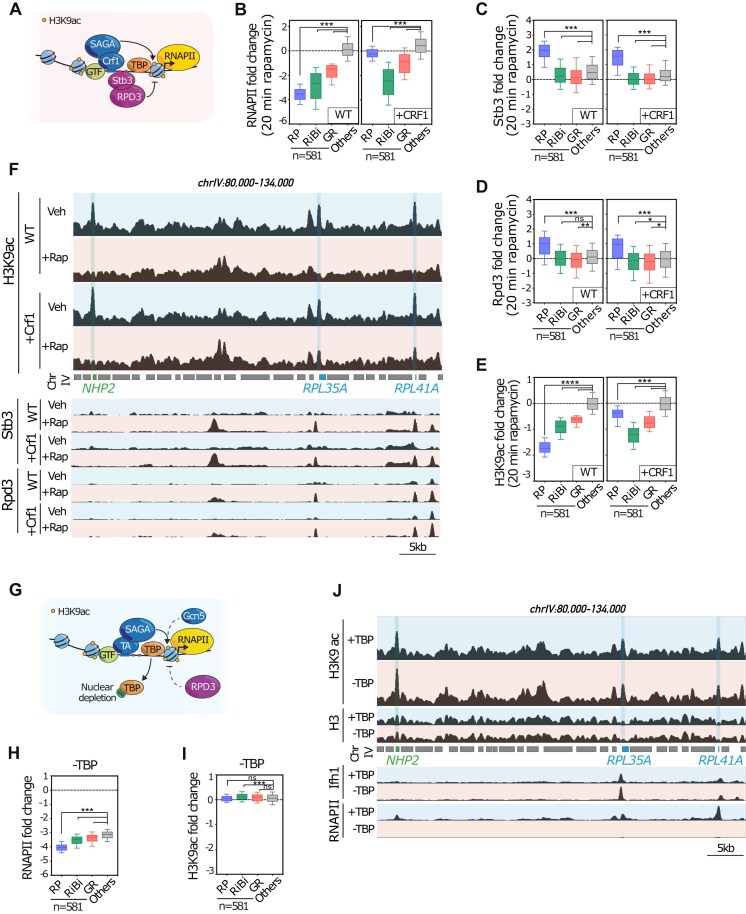
Release of TF, rather than reduction of RNAPII activity, is the first necessary step for rapid deacetylation of H3K9. (**A**) The experimental outline; a specific genetic background allowing maintainance of RP gene transcription following TORC1 inactivation by rapamycin, via the over-expressed and constitutive activator Crf1. (**B**) Box plots showing RNAPII ChIP-seq signals in WT cells (left panel) and the cells with increased Crf1 expression level (right panel), either treated or nontreated with rapamycin (200 nM final concentration) for 20 min. The fold change, calculated as log2 ratio of rapamycin treated versus untreated cells, was assessed for 4966 yeast genes. The average RNAPII signal was quantified from the TSS to the TTS. Genes are categorized based on functional groups: RP, RiBi, GR, and all other genes. Statistical significance for multiple groups was defined by ordinary one-way ANOVA using Dunnett’s multiple comparison test with “others” group as a control (***P< 0.0005). RNAPII ChIP-seq data in WT cells and the cells with increased Crf1 expression level are representative of *n* = 2 ChIP-seq experiments. (**C**) Stb3 occupancy changes in WT cells (left panel) or in *CRF1* overexpressed cells (right panel) following 20 min rapamycin treatment. The average signal of Stb3 was quantified in a window of 500 bp upstream the TSS. The alteration in Stb3 binding was determined by calculating the log2 ratio between rapamycin-treated and nontreated conditions for 4963 genes. Genes are categorized based on functional groups: RP, RiBi, GR, and all other genes. Statistical significance for multiple groups was defined by ordinary one-way ANOVA using Dunnett’s multiple comparison test with “others” group as a control (***P< 0.0005). Stb3 ChIP-seq data in WT cells and the cells with increased Crf1 expression level are representative of *n* = 2 ChIP-seq experiments. (**D**) Same as in (C) but for the Rpd3 occupancy change in WT cells (left panel) or in *CRF1* overexpressed cells (right panel) following 20 min rapamycin treatment. Statistical significance for multiple groups was defined by ordinary one-way ANOVA using Dunnett’s multiple comparison test with “others” group as a control s (*P< 0.05, **P< 0.02, ***P< 0.0005; ns: nonsignificant). Rpd3 ChIP-seq data in WT cells and the cells with increased Crf1 expression level are representative of *n* = 2 ChIP-seq experiments. (**E**) Box plots showing H3K9ac change in WT cells (left panel) and Crf1-overexpressing cells (right panel) following 20 min of rapamycin treatment. The fold change was calculated as log2 ratio of rapamycin treated versus nontreated cells for 5673 yeast genes. The average signal for H3K9ac was quantified in a window of 75 bp centered on the +1 nucleosome. Genes are categorized based on functional groups: RP, RiBi, GR, and all other genes. Statistical significance for multiple groups was defined by ordinary one-way ANOVA using Dunnett’s multiple comparison test with “others” group as a control (****P< 0.0001, ***P< 0.0005). H3K9ac ChIP-seq data in WT cells and the cells with increased Crf1 expression level are representative of *n* = 2 ChIP-seq experiments. (**F**) Genome browser tracks of chromosome IV comparing H3K9ac level (upper panel) and Stb3/Rpd3 bindings (lower panel) in WT cells and the cells with increased Crf1 expression level treated without /with rapamycin for 20 min (Veh versus +Rap). Gene annotations are shown in between the upper and lower tracks. (**G**) Schematic representation of the experimental design to test the effect of transcriptional shut down on H3K9 acetylation; TBP fused to the rapamycin-binding domain FRB and TBP was rapidly depleted from nucleus by using anchor-away assay system upon 1 μg/ml rapamycin treatment for 30 min. Cells were harvested for cross-linking and H3K9ac ChIP-seq following to rapamycin (or vehicle control) treatment. (**H**) RNAPII changes following 30 min depletion of TBP; fold change was calculated as log2 ratio of rapamycin treated versus nontreated cells for 4966 yeast genes. The average signal for RNAPII was quantified and plotted as in panel (B) in the absence (−TBP) and presence of TBP (+TBP). Statistical significance for multiple groups was defined by ordinary one-way ANOVA using Dunnett’s multiple comparison test with “others” group as a control (***P< 0.0005). RNAPII ChIP-seq data following depletion of TBP are representative of *n* = 2 ChIP-seq experiments. (**I**) H3K9ac changes following 30 min depletion of TBP; fold change was calculated as log2 ratio of rapamycin treated versus nontreated cells for 5673 yeast genes. The average signal for H3K9ac was quantified and plotted as in panel (E) in the absence (−TBP) and presence of TBP (+TBP). Statistical significance for multiple groups was defined by ordinary one-way ANOVA using Dunnett’s multiple comparison test with “others” group as a control (***P< 0.0005; ns: nonsignificant). H3K9ac ChIP-seq data following depletion of TBP are representative of *n* = 3 ChIP-seq experiments. (**J**) Snapshots of genome browser tracks showing a region of chromosome chromosome IV for H3K9ac, H3, Ifh1, and RNAPII ChIP-seq (from upper to lower panels, respectively) in TBP nuclear-depleted (−TBP) or nondepleted (+TBP) cells. Gene annotations are shown between the tracks.

Release of the TA Ifh1 and the binding of the repressor Stb3 have both been described to be detectable at 5 min after stress at RP gene promoters [34, 68]. To gain insight into the interplay between these two processes, we took advantage of a specific genetic background that enables the maintenance of transcription at RP genes even under stress conditions, due to the overexpression of Crf1, a paralog of Ifh1 (Fig. 4A and B). We showed previously that Crf1 can act as a constitutive TA of RP genes, probably because it lacks the C- and N-terminal regions of Ifh1 that are essential for its release from RP gene promoters upon stress [29]. Thus, in a Crf1-overexpressing strain, deleted for the *IFH1* gene, RiBi and GR gene transcription are strongly downregulated following TORC1 inhibition, while RP genes are only slightly affected (Fig. 4B). Moreover, we observed that Stb3 and Rpd3 are actively recruited to RP gene promoters in Crf1-overexpressing cells following rapamycin treatment (Fig. 4C, D, and F). Remarkably, though, H3K9ac is only weakly affected at RP genes in Crf1-overexpressing cells (Fig. 4E and F), indicating that recruitment of Stb3 and Rpd3 alone is not sufficient to deacetylate histones at the +1-nucleosome, if a TA remains bound to the promoter. These findings strongly imply that release of Ifh1 is a key event in down-regulation of H3K9ac at RP gene promoters following stress in WT cells, whereas Stb3 acts in a second step required to promote robust H3K9 deacetylation through the recruitment of Rpd3.

Our data agree with findings from previous studies suggesting that dynamic binding of TRs and activators are the key events in regulation of the acetylation state at promoters through recruitment of HAT and HDAC complexes [24]. Nevertheless, experiments described above cannot rule out the possibility that activity of HATs at promoters is itself dependent upon transcription. This is indeed what was recently proposed by Martin *et al.* [69], who reported that transcription is required to expose histone tails in a manner compatible with their acetylation, and thus that TF recruitment of HATs is not by itself sufficient for promoter nucleosome acetylation. However, this conclusion was based upon studies in which RNAPII was inhibited with a metal chelator, 1,10-phenantroline (1,10-pt), which is known to also have profound effects on TF activity [70, 71].

To dissociate the function of TFs in HAT recruitment and/or activation from transcription itself, we chose to rapidly deplete TBP from the nucleus using the anchor-away method ([28]; Fig. 4G). This caused a rapid (30 min) and strong decrease in RNAPII transcription following anchor activation (by rapamycin addition), as expected ([28]; see Fig. 4H and J), yet, remarkably, did not induce any change in H3K9ac levels at promoters (Fig 4I and J). Importantly, we also observed that Ifh1 remained bound to the RP gene promoters following TBP depletion (Fig. 4J and Supplementary Fig. S4), indicating that PIC assembly, but not TF binding, is rapidly impaired by TBP depletion. Our data corroborate previous observations demonstrating that H3K9ac is preserved following the inhibition of transcription [72, 73]. Taken together, these results show that despite a high correlation between H3K9ac and transcription observed in living cells, the rapid deacetylation of the +1-nucleosome cannot be explained by the rapid decrease in transcription observed upon stress. In other words, transcription is not causally related to H3K9 acetylation at promoter nucleosomes, though the two are clearly correlated under normal physiological conditions.

## Discussion

In this study we explored causal relationships between acetylation of histone H3K9 at the +1-promoter nucleosome, the binding of TAs and repressors, and RNAPII transcription initiation. We focused on a group of GR genes where discovered that H3K9ac levels are the most strongly regulated, displaying the highest levels of H3K9ac genome-wide during rapid growth and the lowest levels following stress induction. In a series of functional studies employing deletion mutants or a rapid nuclear depletion approach, we uncovered causal relationships and dependencies between repressor and activator binding, as well as PIC assembly and transcription, that determine the level of H3K9ac at these promoters as a function of growth conditions.

**Figure 5.**
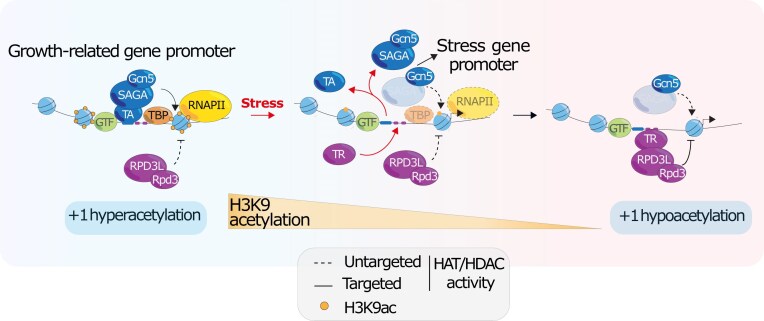
Multiple acetylation states are orchestrated by two-step mechanism driven by the dynamic binding of Transcriptional Repressors (TRs) and activators (TAs).The dynamic level of H3K9ac at GR gene promoters in yeast is regulated by SAGA complex through a two-step mechanism orchestrated by the dynamic binding of both TAs and TRs. The release of TFs from the promoter of GR genes upon stress is a prerequisite and sufficient to allow rapid deacetylation of the +1 nucleosome. Binding of TRs is essential to reach the hypoacetylated state to establish a hypoacetylated state not permissive for transcription. Note that the SAGA complex, which is associated with the untargeted activity of Gcn5, is shown in transparent form to emphasise that we don’t know whether Gcn5 acts alone in this context or is embedded in the SAGA complex.

Based upon our results, and those of previous studies, we propose a model (Fig. 5) wherein multiple H3 acetylation states at a large group of RP, RiBi, and GR genes are orchestrated by a two-step mechanism, driven by the dynamic binding of TRs and activators, that operates independently from PIC assembly or RNAPII binding and transcription. The data show that H3K9ac at the promoters of GR genes is largely driven by SAGA under normal growth conditions and that the release of TAs from the promoter is a sufficient prerequisite to allow rapid deacetylation of the +1-nucleosome. Furthermore, we also show that the binding of TRs (Dot6/Tod6/Stb3) is essential to reach the hypoacetylated state associated with strong silencing of these genes following stress. This dual ability of GR gene promoters to exhibit both hyper- and hypo-acetylation may also help to expand their range of expression levels and more finely tune transcriptional output for optimal growth in widely differing environmental conditions. Consistent with this view, cells lacking one or more of the repressors, or overexpressing Crf1, a constitutive activator of RP genes, display reduced viability and growth under certain conditions [29, 34, 54, 74]. Because RiBi is the most energy-intensive process undertaken during rapid cell growth, and tightly regulated according to nutrient availability [75–77], it seems reasonable to assume that there will be strong selective pressure for tight regulation of this process, beginning at the transcriptional level.

The nuclear levels of Dot6/Tod6, Stb3, Sfp1, and Crf1 have been shown to be directly regulated by their phosphorylation state, which controls their nuclear export or import in a manner dependent on TORC1 and/or PKA pathway activity, whereas Ifh1 is regulated by its sequestration in the nucleolus in a manner also dependent upon growth conditions [67, 68]. Importantly, phosphorylation mutants or truncated alleles of these TFs that maintain their nuclear concentration or prevent their sequestration under stress conditions are sufficient to maintain their activity [29, 34 ,78], consistent with the fact that the available nuclear pools of TFs, more than local promoter architecture in response to stress, is the key determinant of GR gene activity. Such a nuclear concentration-dependent mechanism could facilitate a rapid and coordinated response at GR genes.

As noted above, the strong correlation between promoter H3K9ac and transcription observed during the regulation of these GR genes is in stark contrast to the relatively weak requirement of either SAGA or H3K9ac for their full expression under favorable growth conditions [66]. This finding begs the question of why cells boost H3K9ac at these genes during rapid growth and then employ an elaborate system of repressor-mediated HDAC targeting to strongly down-regulate H3K9ac under stress conditions, to achieve their full repression. Importantly, Gcn5, Rpd3, Stb3, and Dot6/Tod6 are critical for cell survival, chronological lifespan, and stress adaptation, suggesting that efficient and coordinated deacetylation of H3K9 is a key process in cell physiology. Consistent with this view, several studies have convincingly shown that deacetylation of GR gene promoters is essential for metabolism and stress adaptation [61, 79–81]. These data suggest that robust H3K9ac regulation prepares cells to respond better to repeated stress conditions or to recover expression more rapidly after stress withdrawal.

H3K9ac has been described to be highly correlated with other histone modifications, suggesting that the mechanism we describe here could apply more generally (Supplementary Fig. S1O). However, specific combinations of histone marks and histone variants are likely to fine-tune gene expression and may become uncoupled to achieve a wider range of expression. Interestingly, it has been reported that acetylation of H4K5 and H4K12 (targets of Esa1) appear to be less dynamically acetylated than H3K9 [61, 82]. It will be interesting to explore further H4ac states following stress, particularly given the strong redundancy with H3K9ac for expression of most genes under rapid growth conditions, as well as the strong dependency of RiBi genes on the NuA4 HAT Esa1 for their full expression [66]. For example, H3K4me3, commonly considered a mark association with gene activation, does not decrease at RP genes after stress, whereas it does at RiBi genes, highlighting the complexity of the regulatory mechanisms in which histone modifications are implicated [82]. Therefore, the conclusion of our study is limited to H3K9ac at the +1 nucleosome of GR genes. Other acetylation marks at promoters or in coding regions are likely to be regulated by different mechanisms, as recently proposed [69].

It may seem surprising that nontargeted HDAC activity, or at least one independent of Stb3/Dot6/Tod6, is sufficient to rapidly induce a reduction in H3K9ac following the release of TFs at GR genes. However, this finding is fully consistent with an elegant experiment performed more than two decades ago, which demonstrated that the hyper-acetylated state induced by artificial DNA tethering of the HAT-associated TA VP16 can be reversed by the untargeted activity of Rpd3 in only ∼1.5 min after the release of the tethered hybrid protein [24]. Nevertheless, we note that binding of TRs (Dot6/Tod6/Stb3) remains essential to reach a hypo-acetylated state. The fact that GR gene promoters need to be specifically hypo-acetylated upon stress to completely abolish their expression suggests that a basal level of acetylation, dependent on a constant balance between HDAC and HAT activity, might be sufficient to maintain a low level of transcription. This notion fits with a recent model suggesting that the majority of RNAPII promoters in yeast exhibit a basal level of acetylation and are constitutively expressed at a low level without requiring a specific activator [14]. However, it is important to note that this observation does not imply that these genes are stably expressed and remain unregulated under all or even most conditions.

Pursuant to this last point, we believe that the present study highlights the importance of examining global properties of gene regulation under varied physiological conditions. For example, it is well known, but perhaps underappreciated, that a large fraction of the genome is either up-regulated or down-regulated following many different types of stress (e.g. heat shock, osmotic shock, and various forms of nutrient deprivation), and that a significant fraction of this response is common to multiple forms of stress. Even nutrient quality and quantity can have dramatic effects on cell growth rate, which is very tightly linked to the cell’s investment in RiBi. This in turn has important implications for the expression of many other proteins, whose levels will be inversely related to growth rate (see [83, 84] and references therein for a discussion of growth laws). Gene regulation is thus remarkably pervasive in yeast, even for so-called “housekeeping” functions.

Results reported here and elsewhere [45, 65, 66, 85, 86] also underscore the importance of using multiple methods to characterize TF and coactivator or corepressor binding. For example, we showed here (and previously [45]) that ChIP-exo [13, 14] is unable to detect functional Sfp1 binding at the large suite of RiBi genes, as well as many other GR genes. Similarly, another key regulator, Msn2, also a C2H2 zinc finger TF, was only detected at two promoters by ChIP-exo, yet Msn2 binds to and regulates numerous promoters across the genome under stress conditions ([87–90]; see Supplementary Fig. S2A). In contrast to the low level or absence of detection of any HAT co-factor or TF at RiBi genes by ChIP-exo [13, 14], we noticed that 276 out of the 384 proteins used for these meta-analyses were found to have at least one significant binding site on an RP gene promoter. The high transcription rate of the RP genes and their large NDRs, two parameters known to increase the detection of false positives, may also contribute to the discrepancy in ChIP-exo detection of the same factor at different classes of promoters. We also note that approximately half of the TFs (including the ones regulating large transcription programs such as Dot6/Dot6, Xbp1, or Hac1) were not examined in the Mittal *et al.* or Rossi *et al.* studies ([13, 14]; Supplementary Fig. S2F) and the binding of TFs, as well as gene expression, were not tested under a wide panel of growth conditions that might be expected to reveal different binding or expression patterns. Furthermore, the *in silico* analysis conducted by Mittal *et al.* [13] failed to detect the significant enrichment of the PAC motif, which is present more than one hundred times in the UNB group (Supplementary Fig. S2G), suggesting that other TFs or repressors may also have been missed by this analysis. Therefore, despite the impressive quantity and quality of the ∼400 ChIP-exo datasets, the general conclusion that 80% of genes are constitutively expressed [13, 14] must be approached with caution. Taken together, these findings suggest that alternative methods to ChIP must be used to properly characterise the proteins recruited to promoters of RiBi genes and most of the GR genes. However, we would also like to point out that although ChEC-seq may be more sensitive than ChIP, there are technical issues with distinguishing signal from noise that need to be carefully considered when evaluating the results [65, 91 ,92]. Furthermore, in the current ChEC-seq protocol, live cells are incubated in a buffer without glucose or amino acids prior to micrococcal nuclease activation. This stress condition could alter the binding of cofactors that are redistributed across the genome upon stress. All the above strongly suggests that incisive promoter analysis will require the incorporation of data from both ChIP-exo and ChEC-seq localization experiments, ideally carried out under a variety of growth conditions and genetic backgrounds, backed up by functional analyses employing either mutants or rapid depletion/degradation approaches.

In conclusion, we uncover a complex and robust regulatory mechanism by which the dynamic binding of activators and repressors, in response to growth and stress signals, determines the level of H3K9ac at a large set of GR genes. Many of these genes encode either for RPs or proteins involved in RiBi, which are often associated with “housekeeping” functions and the absence of important transcriptional regulation. We hope that the present study will help to highlight the elaborate and varied regulatory mechanisms that control transcription of these genes, whose regulation plays a key role in balancing the energy economy of the cell [77, 93].

## Supplementary Material

gkaf276_Supplemental_Files

## Data Availability

All deep-sequencing data sets have been submitted to the NCBI Gene Expression Omnibus under accession number GSE268170. UCSC Genome Browser session: https://genome.ucsc.edu/s/sevil/GSE268170_BIGWIG%20FILES

## Notes

Present address: Department of Medical Biology, Faculty of Medicine, Pamukkale University, 20160, Denizli, Turkey

Present address: Department of Biological Chemistry and Molecular Pharmacology, Harvard Medical School, Boston, MA02115, United States

present address: molecular, cellular and developmental biology unit (mcd), centre de biologie intégrative (cbi), université de toulouse, cnrs, ups, 31062toulouse, france
