# Supplementary material for: A two-step regulatory mechanism dynamically controls histone H3 acetylation by SAGA complex at growth-related promoters
Source: Nucleic Acids Res. 2025 Apr 10;53(7):gkaf276. doi: 10.1093/nar/gkaf276 (PMC11983098; doi:10.1093/nar/gkaf276)
Supplement: gkaf276_Supplemental_Files [file gkaf276_supplemental_files.zip › sup_figure_legend_20250228.pdf]

Supplementary Figure S1

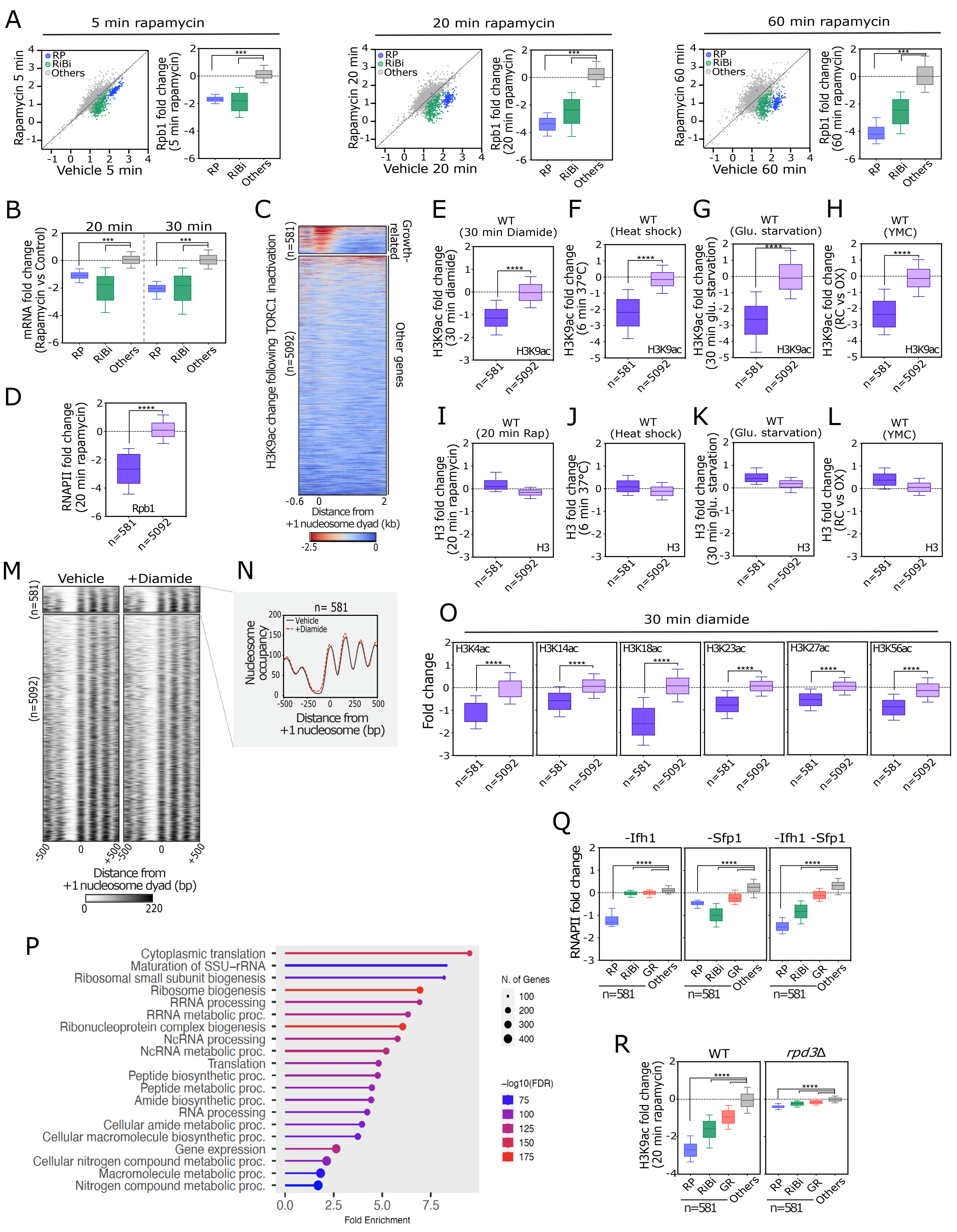

Supplementary Figure S1 (related to Figure 1)

(A) Scatter plots comparing RNAPII binding (as measured by Rpb1 ChIP-seq) in WT cells treated with rapamycin (Y-axis) or vehicle (X-axis) for 5 min (left panel), 20 min (middle panel) and 60 min (right panel). Each dot represents a gene (5092 in total) and genes are color-coded according to functional groups as RP genes (blue), RiBi/RiBiL genes (red) RPGs and all other genes (grey). For RNAPII, the average signal was quantified from the TSS to the transcription termination site (TTS). The scale for both the X-axis and the Y-axis is log10.
(B) Box plots showing mRNA change following either 20 minutes (20 min) or 30 minutes (30 min) of rapamycin treatment. Fold change in mRNA level was calculated as the log2 ratio of treated versus non-treated cells at yeast genes categorized as RP (blue), RiBi (green), GR (red) or all other genes (gray) (data obtained from Urban et al. 2007 (PMID: 17560372)).
(C) Heat maps showing change in H3K9ac ChIP-seq signals for growth-related genes (n= 581) and other genes (n=5092) upon 20 min rapamycin treatment in WT. Heat map is sorted by increasing log2 fold change RNAPII signal upon rapamycin treatment for the same experiment. H3K9 log2 fold change signal change for a window of -600 to +2000 bp relative to +1 nucleosome are displayed (X-axis). H3K9ac ChIP-seq data are representative of n=2 ChIP-seq experiments. The list of 581 identi-fied as rapidly and strongly deacetylated following TORC1 inhibition at RNAPII promoters in n=2 replicates is indicated in Supplementary Table S1 and the list of 86 genes that appears only deacety-lated at their coding region is indicated in Supplementary Table S3.
(D) Box plots showing RNAPII change in WT cells treated or non-treated with rapamycin for 20 min for indicated gene categories. The change in RNAPII calculated as log2 fold change between the signal in rapamycin-treated vs. non-treated cells. Statistical significance of difference between two groups was evaluated using the Mann-Whitney test (****P<0.0001). RNAPII ChIP-seq data are representative of n=2 ChIP-seq experiments.
(E, F, G, H) Box plots showing H3K9ac change in WT cells treated or non-treated with Diamide for 30 min (E) (data obtained from (83)) or during heat shock at 37°C for 6 min (F) (data obtained from (13)), glucose starvation (G) (data obtained from (61)) or between the oxidative and charging phas-es of the metabolic cycle (H) (data obtained from (80)). Statistical significance of difference be-tween two groups was evaluated using the Mann-Whitney test (****P<0.0001).
(I, J, K, L) Box plots showing H3 change in WT cells treated or non-treated with rapamycin for 20 min (I) or during heat shock at 37°C for 6 min (J) (data obtained from (13)), glucose starvation (K) (data obtained from (61)) or between the oxidative and charging phases of the metabolic cycle (L) (data obtained from (80)).
(M) Map showing nucleosome occupancy obtained by Mnase digestion of genes for growth-related genes (n= 581) and other genes (n=5092) in WT cells treated (+ Diamide, right) or non-treated (Vehicle, left) with diamide for 30 min (data obtained from (83)). Mnase-seq Signal for a window of -500 to +500 bp relative to +1 nucleosome are displayed (X-axis).
(N) Average Mnase-seq signal centered on the +1-nucleosome dyad across growth-related genes (n= 581). n WT cells treated (+ Diamide, right) or non-treated (Vehicle, left) with diamide for 30 min (data obtained from (83)). Signal change for a window of -500 to +500 bp relative to +1 nucleosome are displayed (X-axis).
(O) Box plots showing change of acetylation at lysine 4, 14, 18, 23, 27, 56 residues on histone H3 following 30 min diamide treatment in WT cells (data obtained from (83)). The change in acetylation at different lysine residues on histone H3 was calculated and plotted as in (F).
(P) Gene Ontology (GO) enrichment analysis for the genes in 581 promoters was performed using ShinyGO version 0.80 (http://bioinformatics.sdstate.edu/go/). The genes are categorized based on biological processes. The spot size represents the number of genes corresponding to regulated proteins and spot color indicates FDR as p-values. X-axis denotes the enrichment factor of differen-tiated proteins.
(Q) Box plots showing H3K9ac change following either single or double depletion of Sfp1 and Ifh1. Fold change in RNAPII signal was calculated as the log2 ratio of nuclear-depleted versus non-depleted cells at 4966 yeast genes categorized as RP (blue), RiBi (green), GR (red) or all other genes (gray). Statistical significance for multiple groups was defined by ordinary one-way ANOVA using Dunnett's multiple comparison test with the 'other' group as a control (***P<0.0005). RNAPII ChIP-seq data following either single or double depletion Sfp1 and Ifh1 are representative of n=2 ChIP-seq experiments.
(R) Box plots showing the change in H3K9ac ChIP-seq signal in WT and rpd3Δ cells upon rapamycin treatment. The change in H3K9ac calculated as log2 ratio of cells treated or non-treated Vehicle, left. H3K9 ChIP-seq data are representative of n=2 ChIP-seq experiments.

Supplementary Figure S2

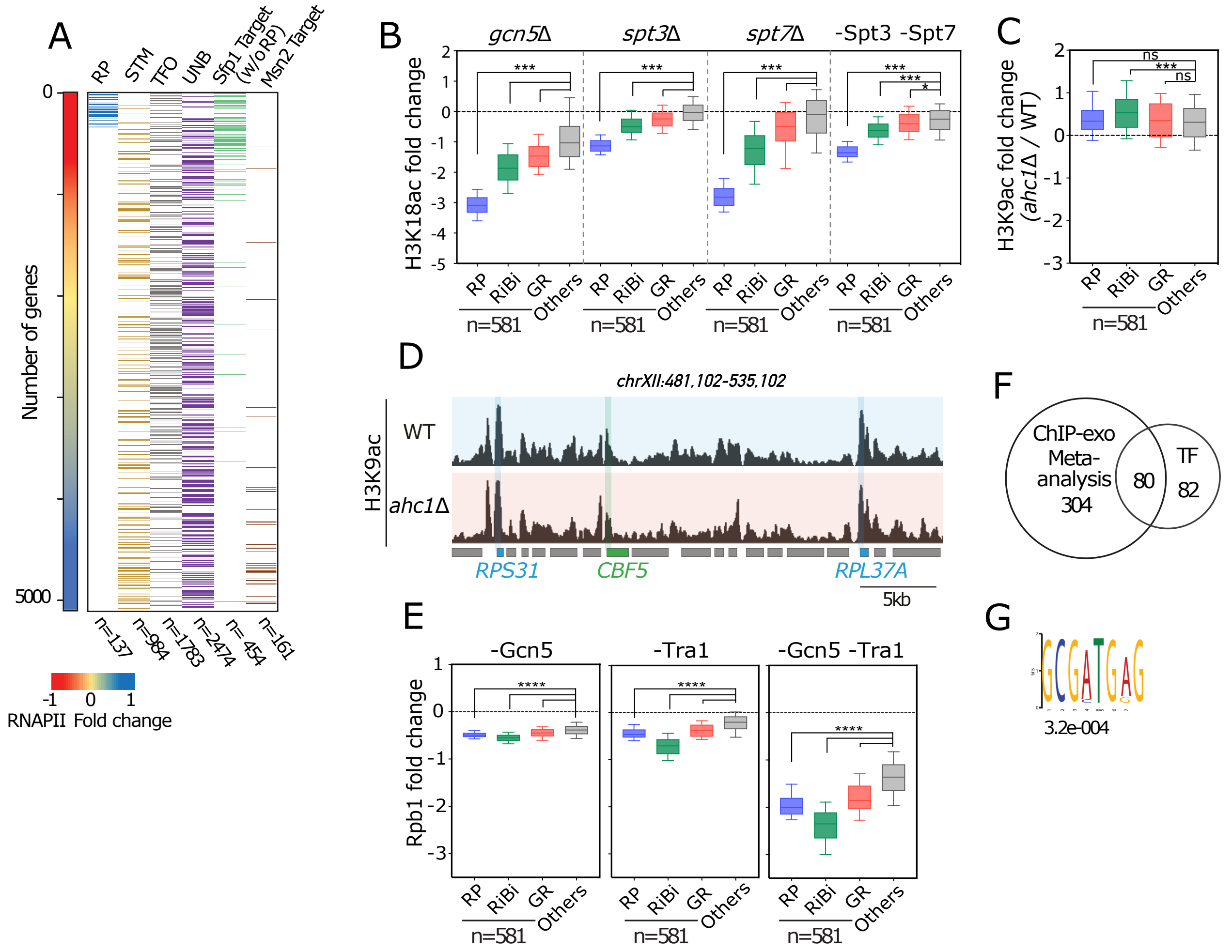

Supplementary Figure S2 (related to Figure 2)

(A) Heatmaps showing different functional gene categories as RP (blue), STM (TF-bound with major co-factor assemblies SAGA, TUP, and/or Mediator/SWI-SNF; brown), TFO (TF-bound, but lacking STM factors; black), UBN (unbound by TFs). (Sfp1-targets (green) and Msn2-targets (gray) defined by ChIP-exo meta-analysis (data from (13)). Heat maps are sorted by increasing log2 fold change RNAPII ChIP-seq signal upon TORC1 inactivation. The number of the genes in each category is shown below the heatmaps.

(B) Fold change of H3K18ac ChIP-seq signal in gcn5Δ, spt3Δ, spt7Δ strains, and in cells depleted for either Spt3 or Spt7 (data from (11)), plotted for the gene categories RP (blue), RiBi (green), GR (red) and all other genes (gray). The change in H3K18ac was calculated as log2 fold change between the signal for WT and mutant cells, or, for the case of Spt3 and Spt7 degradation the signal for cells treated with IAA and vehicle alone (+IAA / DMSO). Statistical significance for multiple groups was defined by ordinary one-way ANOVA using Dunnett's multiple comparison test with 'Others' group as a control (***$P<0.0005$).

(C) Box plots showing the change in H3K9ac ChIP-seq signal in ahc1Δ cells. The change in H3K9ac calculated as log2 ratio in ahc1Δ vs. WT cells. The genes are color-coded according to functional groups as RP (blue), RiBi (green), GR (red) and all other genes (gray). Statistical significance for mul-tiple groups was defined by ordinary one-way ANOVA using Dunnett's multiple comparison test with 'Others' group as a control (***$P<0.0005$; ns: non-significant).

(D) Genome browser tracks showing H3K9ac ChIP-seq read counts on chromosome XII in WT cells and cells lacking the Ahc1 subunit of ADA complex (ahc1Δ). The position of individual genes is shown below the tracks.

(E) Box plots showing Rpb1 change following either single or double depletion of Gcn5 and Tra1. Fold change in RNAPII signal was calculated as the log2 ratio of nuclear-depleted versus non-depleted cells at yeast coding genes categorized as RP (blue), RiBi (green), GR (red) or all other genes (gray) (data obtained from (66)).

(F) Ven-Diagram comparing the number of ChIP-exo experiments used for the meta-analysis of RNAPII promoters (14) with the total number of predicted Transcription Factors in S. cerevisiae.

(G) The PAC motif is significantly enriched at UNB promoters (400 bp region upstream the TSS). MEME analysis (https://meme-suite.org/meme/tools/meme; classic mode, zero or one occurrence per sequence, minimum width 6, maximum width 20) was carried out at all UNB promoters defined by Mittal et al. (13).

Supplementary Figure S3

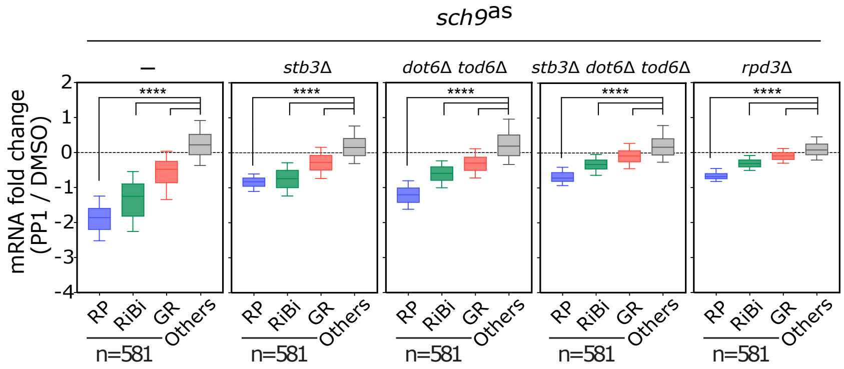

Supplementary Figure S3 (related to Figure 3)
Fold change of mRNA in sch9 analog-sensitive strain (sch9-as) deleted or not for STB3, DOT6, TOD6, RPD3 as indicated, treated or non-treated with PP1 and plotted for the gene categories RP (blue), RiBi (green), GR (red) and all other genes (gray). The change in mRNA was calculated as log2 fold change between the signal for PP1-treated and untreated cells (PP1/ DMSO) ; Statistical signifi-cance for multiple groups was defined by ordinary one-way ANOVA using Dunnett's multiple com-parison test with ‘Others’ group as a control (***P<0.0005). (data from (34)).

Supplementary Figure S4

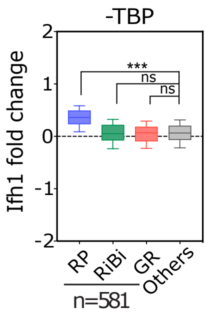

Supplementary Figure S4 (related to Figure 4)
Changes in Ifh1 occupancy upon anchoring away of TBP; fold change was calculated as log2 ratio between TBP-depleted versus non-depleted cells for 4963 genes. The average signal of Ifh1 was quantified in a window of 500 bp centered on the TSS. Genes are categorized and color-coded based on functional groups: RP (blue), RiBi (green), GR (red) and all other genes (gray). Statistical significance for multiple groups was defined by ordinary one-way ANOVA using Dunnett's multiple comparison test with ‘Others’ group as a control (****P<0.0001).
